# Phosphorylation of SNAP-23 regulates its dynamic membrane association during mast cell exocytosis

**DOI:** 10.1242/bio.025791

**Published:** 2017-08-07

**Authors:** Pieu Naskar, Niti Puri

**Affiliations:** Cellular and Molecular Immunology Laboratory, School of Life Sciences, Jawaharlal Nehru University, New Delhi 110067, India

**Keywords:** Exocytosis, Mast cell, RBL-2H3, SNAP-23, SNARE, Allergy

## Abstract

Upon allergen challenge, mast cells (MCs) respond by releasing pre-stored mediators from their secretory granules by the transient mechanism of porosome-mediated cell secretion. The target SNARE SNAP-23 has been shown to be important for MC exocytosis, and our previous studies revealed the presence of one basal (Thr^102^) and two induced (Ser^95^ and Ser^120^) phosphorylation sites in its linker region. To study the role of SNAP-23 phosphorylation in the regulation of exocytosis, green fluorescence protein-tagged wild-type SNAP-23 (GFP-SNAP-23) and its phosphorylation mutants were transfected into rat basophilic leukemia (RBL-2H3) MCs. Studies on GFP-SNAP-23 transfected MCs revealed some dynamic changes in SNAP-23 membrane association. SNAP-23 was associated with plasma membrane in resting MCs, however, on activation a portion of it translocated to cytosol and internal membranes. These internal locations were secretory granule membranes. This dynamic change in the membrane association of SNAP-23 in MCs may be important for mediating internal granule-granule fusions in compound exocytosis. Further studies with SNAP-23 phosphorylation mutants revealed an important role for the phosphorylation at Thr^102^ in its initial membrane association, and of induced phosphorylation at Ser^95^ and Ser^120^ in its internal membrane association, during MC exocytosis.

## Abstract (precis)

**Summary:** The current study has revealed the phosphorylation-dependent dynamic nature of membrane association of SNAP-23 for mediation of different fusion steps in compound exocytosis from mast cells during allergen challenge.

## INTRODUCTION

Mast cells (MCs) are specialized secretory cells that play a crucial role in inflammation and allergic responses (Kalesnikoff and Galli, 2008). During hypersensitivity reactions they are mainly activated by cross-linking of FcεRI-bound IgE by a multivalent allergen (Kraft and Kinet, 2007). This physiological trigger initiates a cascade of events that results in the translocation, docking, and fusion of secretory granules with the plasma membrane leading to the release of inflammatory mediators stored in the secretory granules (Galli et al., 2008; Puri and Roche, 2008). This process proceeds through a transient mechanism of fusion and release, called ‘kiss and run’, and cavicapture for a large proportion of granules in mast cells (Balseiro-Gomez et al., 2016). Further, this secretion involves compound exocytosis where either the vesicles fuse with each other prior to plasma membrane fusion (multivesicular exocytosis) or in a sequential manner, i.e. one after another underneath the plasma membrane (sequential exocytosis) (Lorentz et al., 2012; Pickett and Edwardson, 2006). Recently it has also been shown that in mast cell the granule fusion happens through a supramolecular complex called porosome at the plasma membrane (Balseiro-Gomez et al., 2016; Deng et al., 2010; Hammel and Meilijson, 2012; Jena, 2012).

A class of proteins termed SNAREs (soluble NSF attachment protein receptor) is a known regulator of membrane fusion events involved in exocytosis (Jahn and Südhof, 1999; Lin and Scheller, 2000). Distinct SNARE proteins on vesicles [termed as vesicle SNAREs (*v*-SNAREs)] function by linking up with their cognate SNAREs present on target membranes [termed as target-SNARE (*t*-SNAREs)]. For membrane fusion to occur, the three SNARE proteins from opposing membranes come together to form the minimally required ternary SNARE complex. This involves hydrophobic interactions in the coiled-coil domains of SNARE proteins, and is the essential step in membrane fusion (Poirier et al., 1998; Weber et al., 1998). In a secretory cell, one granule may fuse with another granule to grow into a bigger granule, or with the plasma membrane for exocytosis, by formation of a circular rosette made up of ternary SNARE complexes. The pairing of this SNARE rosette with the plasma membrane is the site defining the fusion pore or porosome where vesicles dock and fuse (Hammel and Meilijson, 2012; Moon et al., 2014).

Formation of the trans-SNARE complex and its temporal and spatial regulation has been studied in MCs (Puri and Roche, 2006). The SNAREs must remain inactive in resting MCs, but on allergen challenge they function rapidly for membrane fusion and degranulation (Hepp et al., 2005). Very little is known about the SNARE function in various fusion steps during exocytosis in MCs. SNAP-23 (synaptosomal associated protein of 23 kDa) plays a key role during regulated exocytosis (Hepp et al., 2005). Our previous studies have shown that SNAP-23 has a basal phosphorylation site at Thr^102^ (Hepp et al., 2005). It is also transiently phosphorylated during regulated exocytosis and the kinetics of phosphorylation is similar to the kinetics of exocytosis (Hepp et al., 2005). However, the precise molecular mechanisms of SNAP-23 function are not known, i.e. what happens immediately after receiving a trigger in a sensitized MC during regulated exocytosis. The early induced phosphorylation of SNAP-23 occurs at two sites (Ser^95^ and Ser^120^) close to the cysteine residues thought to be important for membrane association of SNAP-23. Therefore in this study we decided to check SNAP-23 localization in MCs in resting stage, and immediately after receptor cross-linking, to explore any other important changes that coincide with early transient-induced phosphorylation of SNAP-23 and an initial burst of mediator release in response to a physiological trigger (Hepp et al., 2005). While in the past, one report using permeabilized MCs suggested relocation of SNAP-23 to lamellipodia-like structures (Guo et al., 1998), no study to date has explored SNAP-23 localization under physiological conditions of an allergen challenge leading to receptor cross-linking.

In the current study, as a model system for activation of MCs, we have used cross-linking of DNP-specific IgE sensitized rat basophilic leukemia (RBL-2H3, also referred to as RBL) MCs with the multivalent antigen dinitrophenyl-bovine serum albumin (DNP-BSA), exactly as on MCs of atopic individuals during an allergen challenge (Gould et al., 2003). We found that SNAP-23 is associated with plasma membrane in resting MCs, but after MCs’ activation it moves to internal locations. Then, to study if phosphorylation of SNAP-23 has any role in this aspect, mutants of SNAP-23 that cannot be phosphorylated, or are phospho-mimetic, cloned in EGFP vector for better visualization, were used. This is the first report that highlights the dynamic nature of SNAP-23 membrane association by showing that SNAP-23 changes its location in MCs immediately after receiving a physiological trigger. The study also goes on to reveal the importance of SNAP-23 basal and induced phosphorylation in its initial membrane association, and the dynamic relocations to internal sites on MC activation, respectively. Further elucidation of this pathway of SNAP-23 trafficking would help to generate a comprehensive view of the complex membrane fusion processes that occur in MCs during exocytosis.

## RESULTS

### Activation of RBL MCs by IgE cross-linking partially moves SNAP-23 from plasma membrane to intracellular locations

**Fig. 1.**
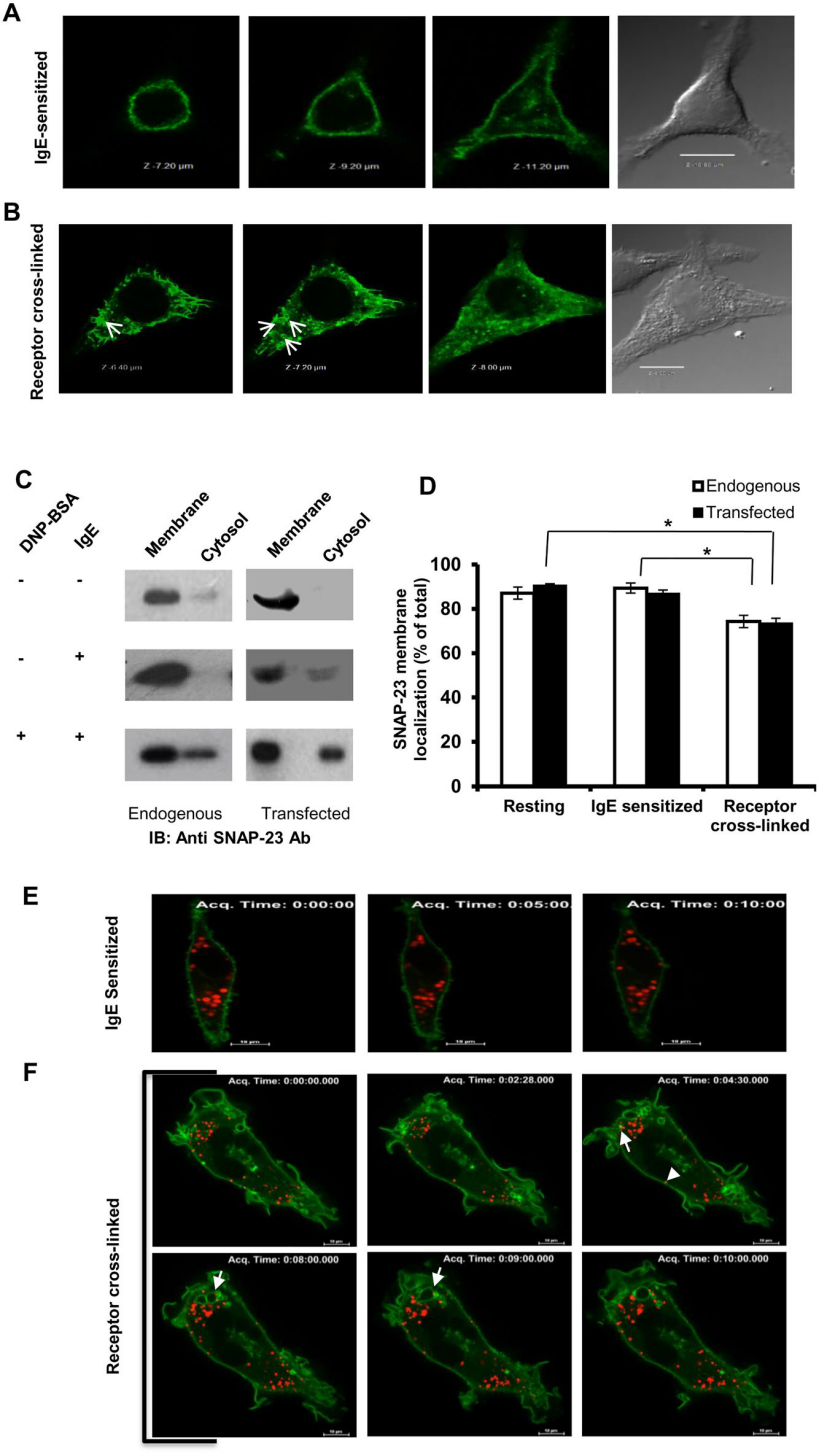
**Membrane localization of SNAP-23 in resting and IgE cross-linked RBL MCs.** (A,B) Representative confocal images showing cross-sections of GFP-SNAP-23 (green) transfected RBL MCs along with DIC images. (A) In resting RBL MCs GFP-SNAP-23 wild type (WT) is associated with plasma membrane (*n*=28). (B) In receptor cross-linked RBL MCs GFP-SNAP-23WT is also seen in internal locations in cytosol (*n*=43). The white arrow heads are indicating GFP-SNAP-23WT in spherical-granule like structures in cytosol. Scale bar: 10 µm. (C) Immunoblot by SNAP-23-specific antibody, showing the membrane and cytosol association of endogenous SNAP-23 and transfected GFP-SNAP-23WT. (D) Quantitative analysis of the immunoblots in C showing a significant decrease in membrane association of SNAP-23 (both endogenous and transfected SNAP-23 WT) in receptor cross-linked. Each data point is mean±s.e.m. of at least three independent experiments (*P≤0.05, student's *t*-test, one-tailed distribution). (E,F) Representative still images from live cell imaging of GFP-SNAP-23WT transfected RBL MCs with Lysotracker Red staining to track lysosomal compartments. SNAP-23 localization at plasma membrane is shown during mock stimulation at three representative time points with no colocalization with Lysotracker Red (E) (derived from live cell). (F) The receptor cross linked panel is showing the snap shots from a representative video (at least 18 videos were captured), depicting SNAP-23 internal membrane localization. White arrows indicate SNAP-23 association with lysosome and arrow heads mark SNAP-23 associated lysotracker. Scale bar: 10 µm.

In the present study, primed RBL-2H3 MCs were stimulated for degranulation by cross-linking of FcεRI (Hepp et al., 2005). The extent of MC degranulation was measured as β-hexosaminidase release at different time points, as shown in Fig. S1A. The degranulation reached a peak between 5-15 min. Using anti-phospho-SNAP-23-Ser^120^ ab (Fig. S1B) it was shown that phosphorylation of SNAP-23 also reached peak at 5 min and persisted until 20 min, indicating that phosphorylation of SNAP-23 is an early and transient event during MC exocytosis (Fig. S1C). Therefore, to capture early changes, all further studies were carried out 10 min after receptor cross-linking. Further, to check whether IgE cross-linking affects the SNAP-23 synthesis/amount we quantified the total SNAP-23 pool at resting, IgE-sensitized, and allergen cross-linked stages of MC by western blotting (for endogenous SNAP-23) and flow cytometry (for EGFP-SNAP-23 transfected RBL MCs) and found no significant difference in the amount of SNAP-23 in any of the stages (Fig. S1D,E). In order to study if these early events lead to any changes in membrane localization of *t*-SNARE SNAP-23 during MC activation, localization of SNAP-23 was studied by confocal fluorescence microscopy. Endogenous SNAP-23 as well as transfected GFP-tagged SNAP-23 was analyzed. Visualization by fluorescence confocal microscopy of the endogenous SNAP-23 (by staining with a SNAP-23 specific antibody, data not shown) and the transfected GFP SNAP-23 revealed a smooth pattern of plasma membrane-localized SNAP-23 in different Z-sections of IgE-sensitized MCs (Fig. 1A). But, 10 min after cross-linking the FcεRI, the cells appeared flatter and the plasma membrane showed ruffles (Fig. 1B, DIC image). It can be seen that SNAP-23 now localized to these plasma membrane ruffles and to some extent on some internal structures (shown by white arrow heads in Fig. 1B). The endogenous and transfected GFP-SNAP-23 were found to behave in a similar fashion both before and after MC exocytosis in terms of their membrane localization (data not shown). Further, to investigate if the internal locations to which SNAP-23 relocated upon IgE cross-linking were internal organelle membranes or cytosolic locations, we performed membrane/cytosol fractionation of resting, IgE-sensitized, and IgE-cross-linked GFP-SNAP-23-transfected as well as untransfected RBL MCs. Quantitative analysis of western blots of the membrane cytosol fractions showed that most [87±2.7% for endogenous and 90±1.5% in case of transfected GFP-SNAP-23 (mean±s.e.m.)] of the SNAP-23 was associated with membrane in resting and IgE-sensitized MCs (89±2.7% for endogenous and 86±1.5% in case of transfected GFP-SNAP-23), however, a small but significant decrease in membrane association (74±2.7% endogenous and 73±2% transfected) of SNAP-23 was observed in MCs 10 min after FcεRI cross-linking (Fig. 1C,D). From this biochemical analysis it is now clear that SNAP-23 relocates to internal cellular locations.

Many of the internal membranes to which SNAP-23 relocated 10 min after receptor cross-linking in MCs were spherical, reminiscent of secretory granules, so we decided to investigate the nature of internal membranes by real-time live-cell microscopic study of GFP-SNAP-23-transfected RBL MCs. These transfected MCs were sensitized with anti-DNP-IgE and lysosomes were marked with Lysotracker Red dye to follow their fate during exocytosis. Before observation in a live-cell imaging system one set of cells were mock stimulated (termed as IgE sensitized) and the other one was stimulated with DNP-BSA. The movie capture was started 2 min after mock or allergen stimulation, and the cells were then observed for 10 min thereafter. Snap shots were extracted from a representative 10 min movie (Movies 1 and 2) obtained from the live-cell imaging system. A good staining of lysotracker dye was observed in both the cases. Like our confocal microscopic study, at IgE-sensitized stage (during mock stimulation) a smooth plasma membrane staining of SNAP-23 (in green) and internal lysosome staining (in red) was seen. During mock stimulation, the IgE-sensitized cells showed slight Brownian movement throughout the 10 min video (Fig. 1E, three time points are shown). But, after allergen addition various dynamic changes in plasma membrane and in lysosomal compartments were seen. At 0 min (which is actually 2 min after stimulation) membrane ruffles can be seen and SNAP-23 is associated with them. Some SNAP-23 starts associating with round vesicles [Fig. 1F (0 min)]. SNAP-23-associated granules harboring lysotracker started appearing at 0 min (2 min after allergen challenge) and they were tracked until 5 min after allergen addition. These vesicles were found to translocate towards plasma membrane (denoted with white arrow and arrow head). In allergen-stimulated cells a bunch of granules with SNAP-23-associated membrane gradually appeared at around 4 min. These green granules were first seen to translocate inside the cell and then started to queue up towards the plasma membrane. During this process at around 7 min some green granules were seen fusing with each other. Ultimately (at 10 min) almost all granules with SNAP-23 ended up in a bigger granule most probably by homotypic fusion. So, this real time imaging shows movement of SNAP-23 to internal vesicle membranes, which may be lysosomal in nature, and fusion of some of these vesicles probably during compound exocytosis from MCs on allergen challenge.

### SNAP-23 moves from plasma membrane to internal lysosomal membranes during regulated exocytosis of MCs

**Fig. 2.**
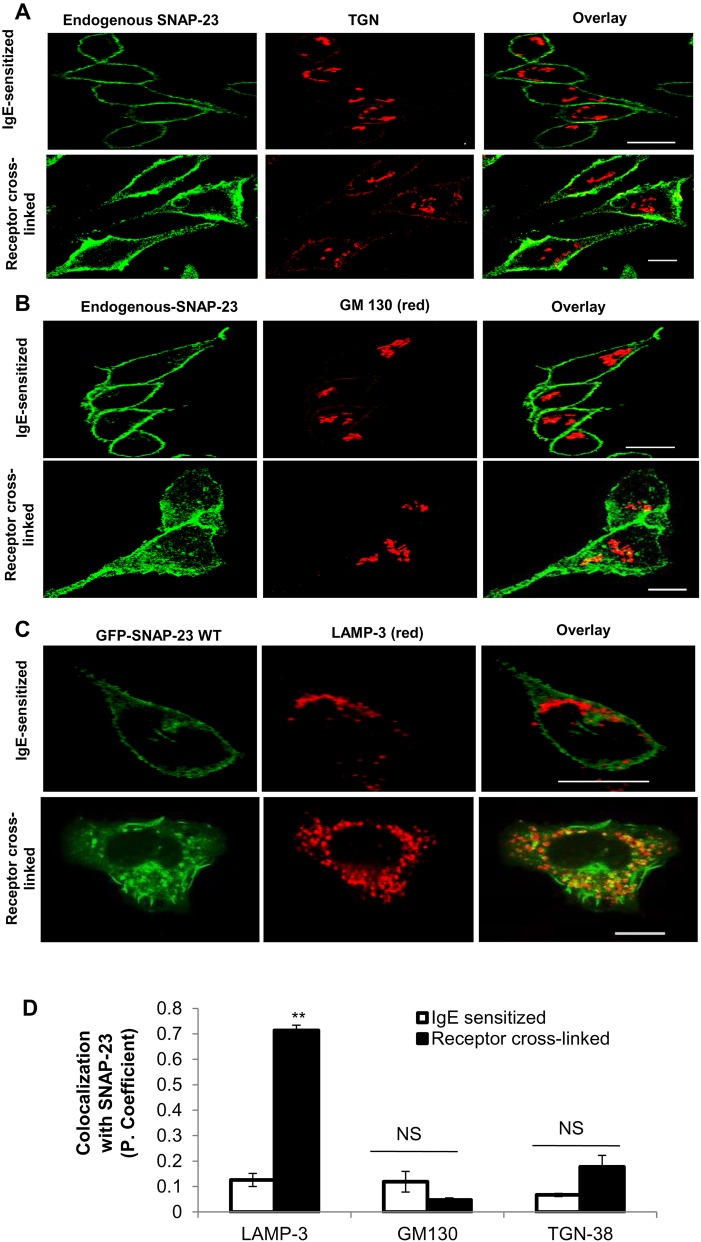
**SNAP-23 shows significant colocalization with lysosomal granule marker LAMP-3 but not with TGN38 and GM130 in allergen-stimulated RBL MCs.** (A,B) The representative confocal images are showing that endogenous SNAP-23 (green, SNAP-23 Ab) does not relocate to the TGN or Golgi membrane [(red, TGN-38 Ab; *n*=71, IgE sensitized; *n*=100, receptor cross-linked) and GM-130 Ab (*n*=100, IgE sensitized; *n*=70, receptor cross-linked)] after receptor cross-linking of MCs. (C) Representative confocal image is showing the GFP-SNAP-23WT (green) co localization with lysosomal granules (red, granule marker LAMP-3-specific Ab). A high amount of colocalization (yellow, overlay) of GFP-SNAP-23WT with LAMP-3 containing granules was observed after receptor crosslinking of MCs (*n*=15). In all resting states SNAP-23 resides on plasma membrane with no colocalization with any internal organelle marker. Scale bar: 10 μm. (D) The graph representing the co-localization of SNAP-23 (both endogenous and transfected) with the counterstained markers (above) in terms of Pearson coefficient. Each data point is mean±s.e.m. of three independent experiments (**P≤0.005, Student's *t*-test, one-tailed distribution).

To confirm the observation obtained from previous experiments we performed immunofluorescence and confocal microscopy. We have studied the association of SNAP-23 with different internal organelles like Golgi apparatus, trans-Golgi network (TGN), and also late endosome/lysosomes. RBL MCs were first stained with SNAP-23-specific antibody and then counter stained with TGN38 [a type I integral membrane protein primarily localized to the TGN (Humphrey et al., 1993)]- and GM130 [Golgi matrix protein of 130 kDa, peripherally associated with the cis-compartment (Nakamura et al., 1997)]-specific antibodies at IgE-sensitized and receptor cross-linked states (Fig. 2A and B, respectively). The confocal microscopy images from Fig. 2A and B showed that SNAP-23 is not associated with Golgi and TGN at IgE-sensitized states of MCs [Pearson coefficient 0.001±0.002 and 0.0357±0.0014 (mean±s.e.m.), respectively, Fig. 2D] and also after receptor cross-linking it does not relocate to these internal organelle membranes [Pearson coefficient 0.15±0.042 and 0.054±0.002 (mean±s.e.m.), respectively, Fig. 2D]. In order to investigate if any SNAP-23 localized to late endosome/lysosomal membranes in activated MCs, LAMP-3 lysosomal membrane marker in MCs was used. GFP-SNAP-23-transfected MCs were counterstained with anti-LAMP-3 antibody. As shown in Fig. 2C, in IgE-sensitized GFP-SNAP-23 expressing MCs SNAP-23 is mainly localized to plasma membrane and LAMP-3 staining is completely internal, and negligible colocalization is seen between the two (Pearson coefficient 0.17±0.001, Fig. 2D). But after receptor cross-linking, transfected GFP-SNAP-23 was found on plasma membrane ruffles and also on internal membranes, a large number of which also showed staining for LAMP-3 (Fig. 2C). So, a very high level of colocalization was obtained between GFP-SNAP-23 and LAMP-3 (Pearson coefficient 0.7±0.03, Fig. 2D). This indicated that 10 min after receptor cross-linking SNAP-23 relocated to internal membranes in MCs, which were LAMP-3-positive and hence lysosomal in nature.

### SNAP-23 relocates to secretory granule membranes during regulated exocytosis in MCs

**Fig. 3.**
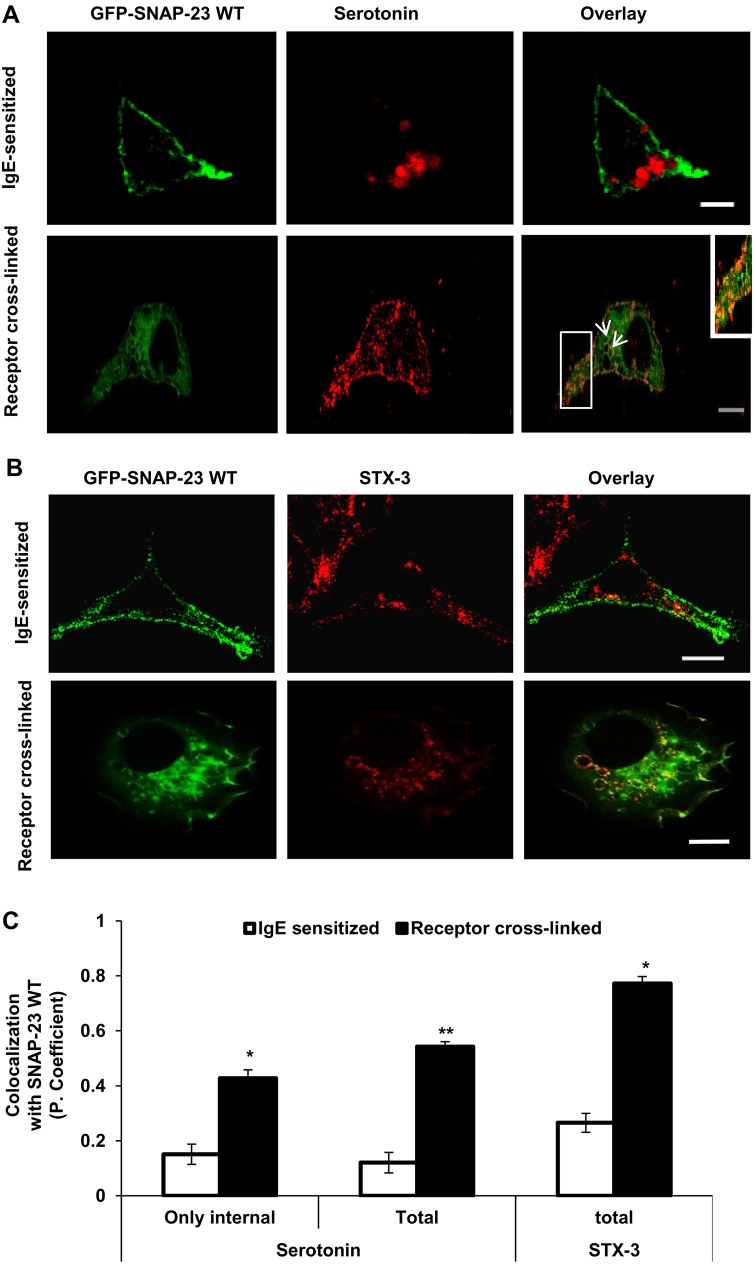
**SNAP-23 shows co-localization with secretory lysosomes in allergen-activated RBL MCs.** (A) GFP-SNAP-23WT (green) relocates to internal serotonin (red, anti-Serotonin Ab) containing granules after MCs receptor cross linking (*n*=20). The inset image shows an enlarged view of over-lapping region indicated by the white box and the white arrows depicting green-SNAP-23-associated granules containing red serotonin cargo within them. (B) In IgE-sensitized MCs GFP-SNAP-23 (green) does not co-localize with STX-3 containing granules (red, STX-3 specific antibody) (*n*=32). But after the allergen cross-linking a significantly high amount of SNAP-23 was found to co-localize with STX-3 containing secretory granules (*n*=42). Scale bar: 10 μm. (C) The graph representing the colocalization of GFP-SNAP-23WT with the counterstained markers (above) in terms of Pearson coefficient. Each data point is mean±s.e.m. of at least three independent experiments (*P≤0.05; **P≤0.005; Student's *t*-test, one-tailed distribution).

Since lysosomes in MCs also have secretory functions, and are therefore referred to as secretory lysosomes (Puri et al., 2003; Puri and Roche, 2008), we wanted to check whether SNAP-23 also relocates to the secretory granule. Rodent MCs are known to harbor serotonin in granules that are lysosomal in nature, and there is a regulated release of serotonin in response to a physiological trigger (Puri and Roche, 2008). We decided to locate serotonin cargo in GFP-SNAP-23-transfected RBL cells by counterstaining serotonin with serotonin-specific antibody in IgE-sensitized and allergen-activated RBL MCs. Syntaxin-3 (STX-3) in the IgE-sensitized MCs, SNAP-23 showed plasma membrane association and serotonin showed a punctate staining pattern in intracellular organelles (Fig. 3A, upper panel) with negligible or no co-localization (0.1±0.018, Fig. 3C). But, 10 min after FcεRI cross-linking, as SNAP-23 localization pattern changed, it showed partial co-localization with serotonin [Pearson coefficient 0.43±0.03 (internal, i.e. excluding plasma membrane), Pearson coefficient 0.54±0.018 (including plasma membrane, total)] (Fig. 3A, lower panel). The white arrows and inset image indicate serotonin punctae surrounded by organelle membranes having GFP-SNAP-23 staining. This indicated that SNAP-23 colocalized to internal secretory granule membranes in MCs on activation by FcεRI cross-linking. Besides, from the literature it is known that *t*-SNARE Syntaxin-3 (STX-3) resides in secretory granule membrane (Puri et al., 2003), so we also looked at STX-3 co-localization, if any, with SNAP-23 in GFP-SNAP-23-transfected RBL MCs by counterstaining these cells with anti-STX-3 ab. In the case of IgE-sensitized MCs, STX-3 is seen on internal granule membranes and does not show any co-localization with GFP SNAP-23 (Fig. 3B, upper panel; Pearson coefficient 0.26±0.03, Fig. 3C) expressed on plasma membrane. After receptor crosslinking, the relocated SNAP-23 showed very high colocalization with STX-3 on almost circular internal secretory granule membranes (Fig. 3B, lower panel; Pearson coefficient 0.77±0.02, Fig. 3C). Together these two results indicate that immediately after receptor crosslinking, SNAP-23 relocates to STX-3 harboring internal organelles which may also enclose the secretory granule cargo like serotonin, and hence are secretory granules.

### Phosphorylation of SNAP-23 is important for its membrane association in RBL MCs

**Fig. 4.**
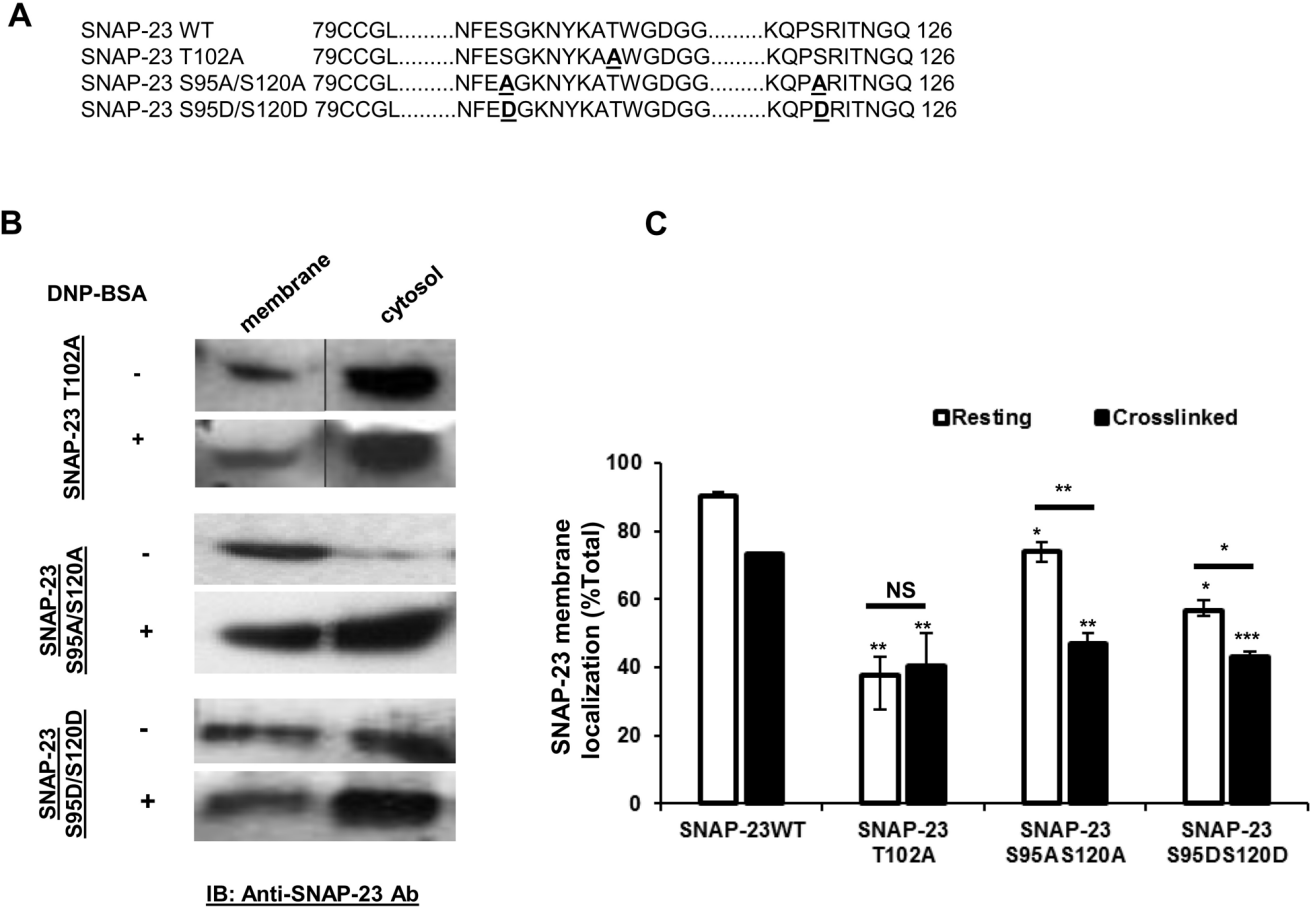
**SNAP-23 phospho mutants and their dynamic membrane association.** (A) Sequence alignment showing the SNAP-23WT and other phospho mutants (T102A, S95A/S120A, S95D/S120D) in the linker region. Protein sequences obtained from their respective DNA sequences by using ‘DNA to Protein translation tool’ (http://insilico.ehu.es/translate/index.php). (B) Immunoblots showing the membrane and cytosol association of GFP-SNAP-23 phospho mutants (by SNAP-23-specific antibody). Lanes are cut from the same blots and repositioned (for GFP-SNAP-23 T102A mutant). (C) The band intensities were quantified by spot denso Alpha EaseFC software. The percentage membrane association of SNAP-23 reveals that the mutation of the above residues affects SNAP-23 dynamic association as well as cytosolic accumulation. Each data point is mean±s.e.m. of three independent experiments (*P≤0.05; **P≤0.005; ***P≤0.0005; NS, not significant; Student's *t*-test, one-tailed distribution).

SNAP-23 lacks transmembrane domain and is thought to associate with the plasma membrane through palmitoylation of its conserved cysteine residues present in the linker region (Vogel and Roche, 1999; and V. Agarwal, P. N., N. P., unpublished data). Our previous studies have identified one basal phosphorylation site, Threonine^102^ (T^102^), close to these cysteines in the linker region of SNAP-23. To explore if phosphorylation of T^102^ has any role in association of SNAP-23 with membranes, T was mutated to A, so that it can no longer be phosphorylated (Fig. 4A). GFP-SNAP-23 T102A-transfected RBL MCs were either mock-stimulated or receptor-cross-linked for 10 min and subjected to membrane cytosol fractionation. Immunoblotting of membrane cytosol fractions of mock-stimulated cells revealed significantly lower association (55-60% decrease) of SNAP-23 T102A mutant with membrane in comparison to wild-type SNAP-23 (Fig. 4B,C). Similar results were obtained for transfected cells activated by receptor cross-linking, with a major portion of mutant SNAP-23 residing in the cytosol (60% in cytosol) (Fig. 4B,C).

Further, the induced phosphorylation sites at S^95^ and S^120^ are also very close to the conserved cysteines in the linker region of SNAP-23. So, to investigate the role of SNAP-23 transient phosphorylation followed by dephosphorylation 20 min after receptor cross-linking in membrane localization of SNAP-23, two kinds of mutants, GFP SNAP-23 S95A/S120A (phospho-negative, that cannot be phosphorylated) and GFP SNAP-23 S95D/S120D (phospho-mimetic, constitutively phosphorylated) (Hepp et al., 2005), were used. The amino acid sequence comparison for these two mutants with wild-type SNAP-23 is shown in Fig. 4A. In case of IgE-sensitized RBL MCs, SNAP-23 S95A/S120A mutant was shown, by membrane cytosol fractionation, to be mainly associated with membrane, though there was a small but significant decrease in membrane association in comparison to wild-type SNAP-23 (decrease from 90 to 73%) (Fig. 4B,C). In the receptor cross-linked stage, the membrane association of SNAP-23 S95A/S120A mutant showed a drastic decrease in comparison to wild-type SNAP-23 (from 73 to 47%), and a major portion of it was cytosolic (Fig. 4B,C).

The phospho-mimetic SNAP23 S95D/S120D mutant mimics the transient phosphorylated state which lasts 5 to 20 mins after receptor crosslinking. In mock-stimulated stage, membrane cytosol fractionation of SNAP-23 S95D/S120D-transfected RBL cells revealed much lower association of SNAP-23 mutant with membrane in comparison to wild-type SNAP-23 (56% in comparison to 90%) (Fig. 4B,C). After activation, there is a further decrease in membrane association, and increase in cytosolic localization, of SNAP-23 S95D/S120D mutant (43% membrane association) (Fig. 4B,C).

### Role of SNAP-23 phosphorylation in its dynamic association with internal membranes during secretory response of MCs

**Fig. 5.**
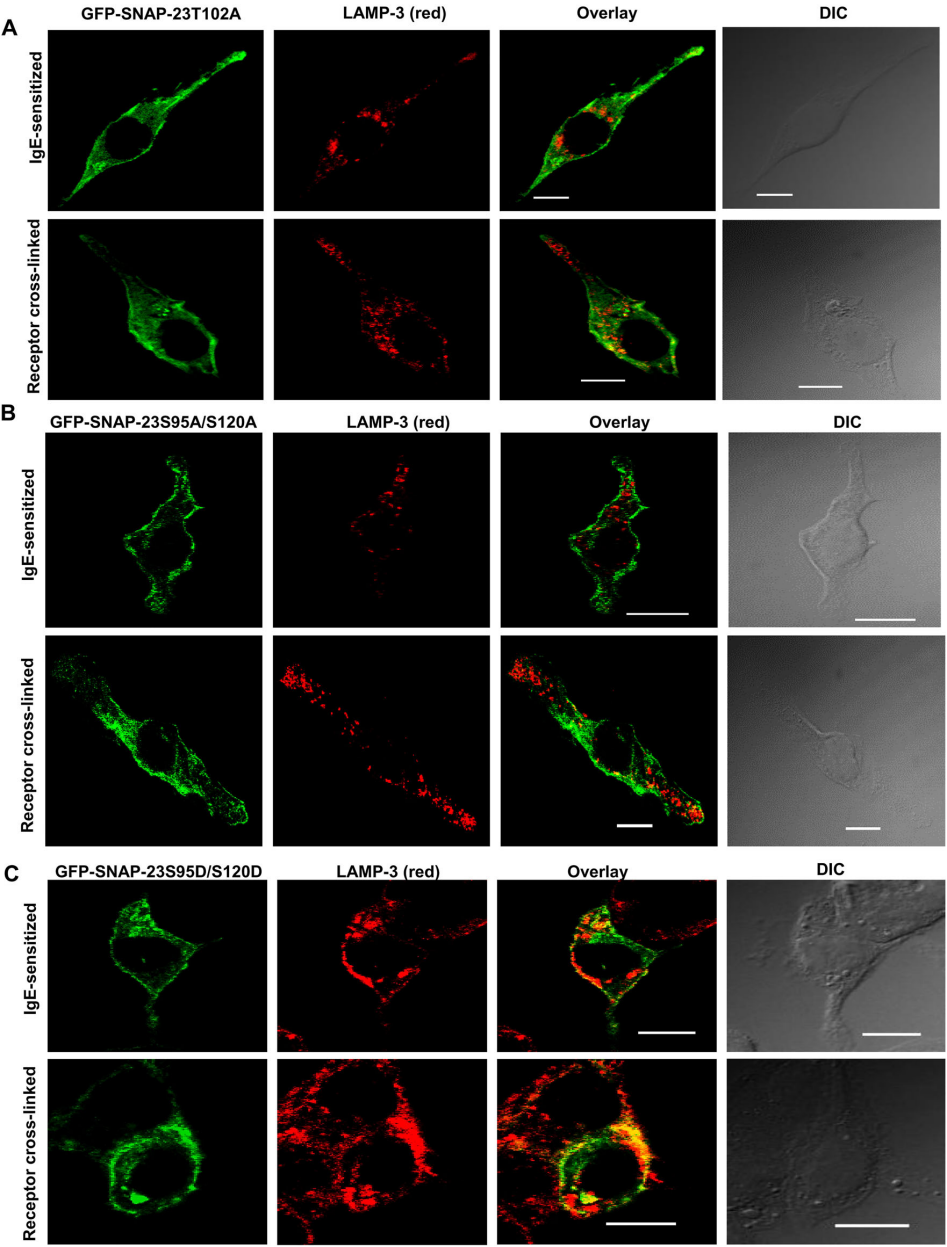
**Effects of mutations at T^102^, S^95^ and S^120^ in membrane association of SNAP-23.** (A-C) Representative confocal microscopy images are showing the GFP-SNAP-23 T102A (*n*=17) and GFP-SNAP-23 S95A/S120A (*n*=34) (green) do not colocalize with LAMP-3 positive granules (anti-LAMP-3 Ab, red) in IgE-sensitized and receptor cross-linked MCs. But GFP-SNAP-23 S95D/S120D shows high level of association with LAMP-3 (*n*=20) in IgE-sensitized and receptor cross-linked MCs as well. Scale bar: 10 μm.

As previous experiments with wild-type SNAP-23 had shown relocation to internal lysosomal membranes on receptor cross-linking, the co-localization of SNAP-23 T102A with lysosomal compartments was also investigated by microscopy after counterstaining transfected cells with LAMP-3-specific antibodies. As shown in Fig. 5A, a lot of intracellular or cytosolic location for transfected SNAP-23 T102A mutant in both IgE-sensitized and receptor-cross-linked stages was confirmed. SNAP-23 T102A mutant also did not show any co-localization with LAMP-3 in resting stage (Pearson coefficient 0.17±0.03), as the wild-type SNAP-23 (Pearson coefficient 0.12±025). But even after receptor cross-linking SNAP-23 T102A does not show any significant co-localization with LAMP-3-positive membranes (Pearson coefficient 0.28±0.06). These results indicate that basal phosphorylation of SNAP-23 at T^102^ may be important for initial association of SNAP-23 with membranes as the membrane association showed a marked decrease even in mock-stimulated or resting stage (Fig. 5A). The phosphorylation of T^102^ may also be important for relocation of SNAP-23 to internal lysosomal membranes on receptor cross-linking as SNAP-23 T102A mutant does not show association with lysosomal membranes on activation and remains stuck in cytosol (Fig. 5A, Table 1).

**Table 1.**
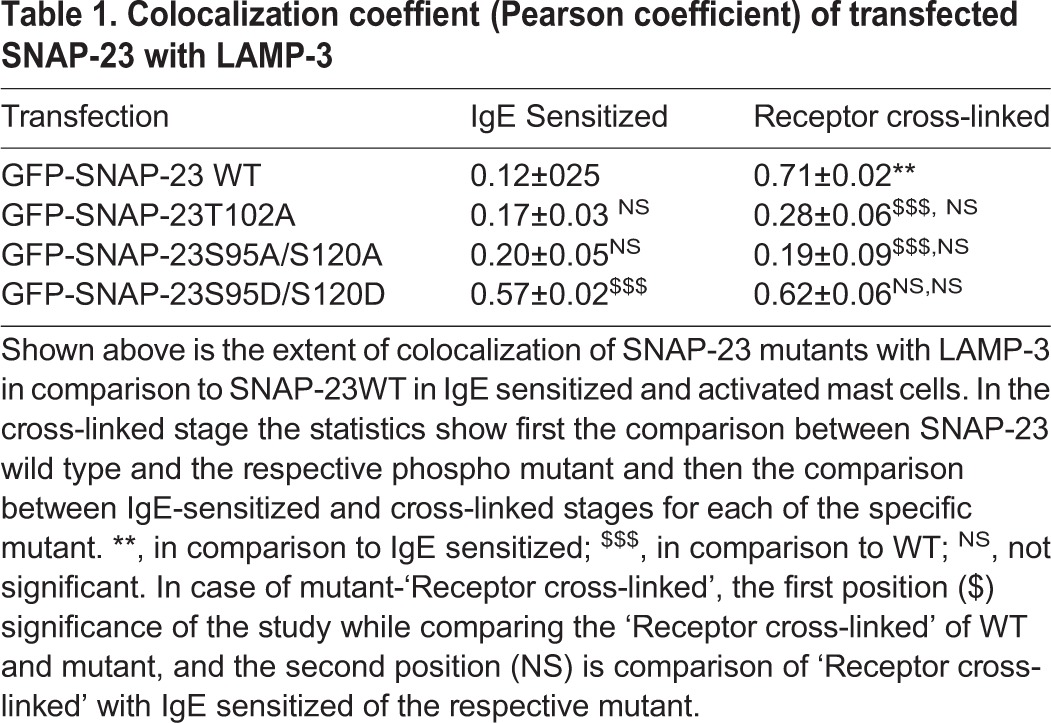
**Colocalization coeffient (Pearson coefficient) of transfected SNAP-23 with LAMP-3**

The increased cytosolic localization of the SNAP-23 S95A/S120A mutant was confirmed by immunofluorescence microscopic study. When the co-localization of this SNAP-23 S95A/S120A mutant with lysosomal compartments in resting or activated MCs was tested, by staining transfected MCs with LAMP-3 Ab, no colocalization of the SNAP-23 S95A/S120A mutant with LAMP-3 was seen at either stage (Pearson coefficient 0.20±0.05 and 0.19±0.09 in IgE-sensitized and receptor-cross-linked MC, respectively) (Fig. 5B, Table 1). This study with the SNAP-23 S95A/S120A mutant, that cannot be phosphorylated, reveals that transient phosphorylation of SNAP-23 at Ser^95^ and Ser^120^ may be important for relocation of SNAP-23 to internal lysosomal membranes, prevented by lack of phosphorylation and leaving SNAP-23 stuck in the cytosol on MC activation.

The increased cytosolic association of the SNAP-23 S95D/S120D mutant was also observed by confocal fluorescence microscopy. Further, when the transfected RBL MCs were co-stained with LAMP-3 ab, a very high colocalization of the SNAP-23 S95D/S120D mutant with LAMP-3 was observed (Pearson coefficient 0.57±0.02), even in mock-stimulated stage. This showed that a large proportion of mutant SNAP-23 was already associated with internal lysosomal membranes rather than with the plasma membrane, as happens in the case of wild-type SNAP-23. On receptor crosslinking, the SNAP-23 S95D/S120D mutant maintains very high colocalization with LAMP-3 (Pearson coefficient 0.62±0.06) (Fig. 5C, Table 1). These results indicate that the transient induced phosphorylation of SNAP-23 at Ser^95^ and Ser^120^ plays a very important role in movement of SNAP-23 to internal locations and then its association with lysosomal membranes.

### Role of SNAP-23 phosphorylation in MC exocytosis

**Fig. 6.**
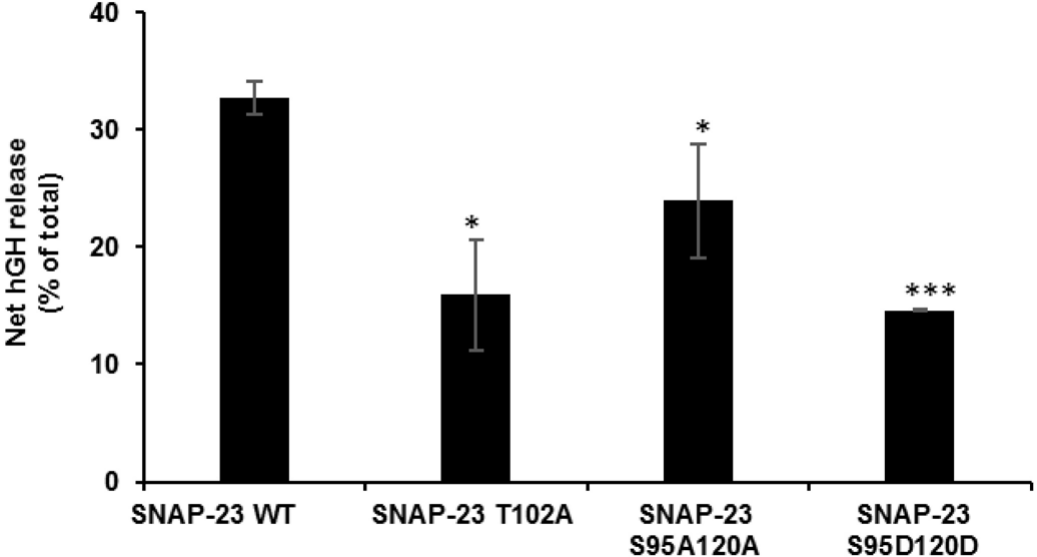
**Expression of phospho mutants affects exocytosis from transiently transfected RBL MCs.** The bar graph shows the extent of exocytosis as net (percent of total) release of hGH secretion reporter from transfected RBL MCs on receptor cross-linking. Transfection with SNAP-23 T102A, SNAP-23 S95A/S120A and SNAP-23 S95D/S120D causes a significant inhibition in net hGH release from transfected RBL MCs in comparison to cells with WT SNAP-23. The data shown are mean±s.e.m. of three independent experiments, and asterisks indicate statistically signiﬁcant differences in hGH release between SNAP-23 WT versus mutant transfected RBL MCs (*P≤0.05; ***P≤0.0005; Student's *t*-test, one-tailed distribution).

SNAP-23 has been shown to play an important role in MC degranulation (Hepp et al., 2005). After observing dramatic effects of mutations in basal and induced phosphorylation sites of SNAP-23 on its membrane association in resting and activated MCs, it was decided to check the effects of these mutations on MC degranulation. For this, the hGH secretion reporter assay [already discussed in Puri et al. (2003) and briefly in the Materials and methods] was performed. The GFP-SNAP-23 phospho mutants and hGH DNA were co-transfected in RBL MCs and their extent of hGH release was assayed at 45 min. In the case of MCs transfected with GFP-SNAP-23 T102A mutant, significant reduction in secretion (∼54% reduction in comparison to wild-type SNAP-23) was seen after allergen cross-linking (Fig. 6). In the case of induced phosphorylation site mutations, hGH release assay of GFP-SNAP-23 S95A/S120A and GFP-SNAP-23 S95D/S120D mutant transfected MCs (separately) showed reduction in secretion (∼30% and ∼57% reduction, respectively, in comparison to wild-type SNAP-23) after allergen cross-linking (Fig. 6). The expression of transfected GFP-SNAP-23 wild type and mutants was also compared to that of endogenous SNAP-23 by immunoblotting the lysates from transfected cells with anti-SNAP-23 Ab (Fig. S2A). In all cases, the level of expression of transfected proteins was about 40% of that of endogenous SNAP-23 (Fig. S2B).

## DISCUSSION

The plasma membrane *t*-SNARE SNAP-23 plays an important role in MC exocytosis (Hepp et al., 2005). SNAP-23 and its ability to form ternary SNARE complexes is required for MC degranulation (Vaidyanathan et al., 2001; Weber et al., 1998), but the exact molecular mechanisms of SNAP-23 function immediately after receiving an activation trigger in a MC are not known except for the concomitant transient phosphorylation of SNAP-23 during regulated exocytosis (Hepp et al., 2005). Our previous studies (Hepp et al., 2005) have shown that SNAP-23 gets phosphorylated at Thr^102^, Ser^95^ and Ser^120^. Among these, the Thr^102^ phosphorylation site remains constitutively phosphorylated and other two are inducible in nature. Further, it is known that impairment of induced phosphorylation results in reduced exocytosis (Hepp et al., 2005). In this study we looked at SNAP-23 localization in MCs immediately after receptor cross-linking, and the role of SNAP-23 phosphorylation in this process. One earlier report using permeabilized MCs reconstituted with purified cytosol from rat brain showed that SNAP-23 relocated from plasma membrane lamellipodia to internal structures within seconds of receiving a calcium trigger (Guo et al., 1998). Our model system for activation of MCs uses cross-linking of DNP-specific IgE-sensitized MCs with the multivalent antigen DNP-BSA mimicking MC activation during an allergen challenge (Gould et al., 2003).

During our microscopic study of SNAP-23, we observed that SNAP-23 membrane association changed dynamically upon allergen trigger and some of it seemed to relocate to intracellular sites. Membrane cytosol fractionation experiments revealed that a majority of SNAP-23 was still membrane-associated but a significant proportion now resided in the cytosol of MCs. So, a proportion of SNAP-23 translocated to internal organelle membranes and a small portion translocated to the cytosol. Earlier in various cell types, *t*-SNAREs and SNARE interacting proteins important for exocytosis were shown to recycle between different internal organelles. Syntaxin-3 and -4 were shown to recycle from plasma membrane to TGN and Rab-11-positive recycling endosomes, respectively, in rat kidney cells (Band et al., 2002; Band and Kuismanen, 2005). In dendritic cells Syntaxin-3 is generally expressed in cytosol, but upon LPS stimulation it translocates to plasma membrane to mediate IL-6 secretion (Collins et al., 2015). After ionomycin stimulation SCAMP-5 relocates to recycling endosomes from Golgi compartments (where it co-localized with Syntaxin-6) and subsequently to plasma membrane during tumor necrosis factor (TNF) secretion from activated macrophages and co-localizes with Syntaxin-4/SNAP-23 (Murray and Stow, 2014). Similarly, SNAP-23 relocation in MCs in response to a physiological trigger may be important for mediating some important fusion steps during MC mediator release.

To specifically define the internal locations to which SNAP-23 relocates on MC activation, we took help from our previous studies showing that a large proportion of MC secretory granules are lysosomal in nature (Puri and Roche, 2008). When we co-stained resting MCs with LAMP-3 antibody almost no co-localization between LAMP-3 and SNAP-23 was observed. But, 10 min after receptor cross-linking a significant amount of co-localization between SNAP-23 and LAMP-3 was observed, showing relocation of SNAP-23 to LAMP-positive internal late endosomal/lysosomal compartments. Similar observations were made in live cell imaging with SNAP-23 and lysotracker as lysosomal marker. We have also shown that LAMP-positive secretory granule in rodent MCs harbor cargo like serotonin or cathepsin D and the *t*-SNARE Syntaxin-3 (Puri et al., 2003; Puri and Roche, 2008). So, we co-stained MC with either serotonin or Syntaxin-3-specific antibodies, respectively, and found no co-localization of plasma membrane-associated SNAP-23 with granule-associated serotonin or Syntaxin-3 in resting MCs, but a very significant co-localization of SNAP-23 with serotonin and Syntaxin-3, 10 min after receptor cross-linking. This shows conclusively that SNAP-23 relocates to membrane of internal secretory granules.

The main questions are how and why does SNAP-23 end up on internal membranes in MCs early after receptor cross-linking. Since some SNAP-23 still remained associated with plasma membrane, only a portion of the total pool relocates to internal membranes. There are two possible mechanisms by which SNAP-23 may relocate to internal membranes. One possibility is that it dissociates from plasma membrane, enters cytosol, and again binds to internal lysosomal membranes. In fact, we do see a small but significant increase in SNAP-23 in the cytosolic fraction 10 min after receptor cross-linking. This could be due to SNAP-23 being on its way to relocate to internal membranes. There are reports showing that the extent of palmitoylation determines the differential membrane association of a protein (Greaves and Chamberlain, 2011). It may be that the induced phosphorylation of SNAP-23 during exocytosis (Hepp et al., 2005) regulates the palmitoylation of SNAP-23 so that it dissociates from plasma membrane and associates with lysosomal membranes. The other possibility is that immediately after stimulation one or a few granules release their content via the ‘kiss and run’ mechanism and quickly SNAP-23 relocates with this granule. It has been reported earlier that MCs exhibit both ‘kiss and run’ and cavicapture types of transient granule-plasma membrane fusion to maintain their granularity and to retain the capacity of undergoing repeated exocytosis (Balseiro-Gomez et al., 2016; Cohen et al., 2012). Further, a recent study involving atomic force microscopy (AFM) and transmission electron microscope (TEM) detailed the capture of a typical porosome on activated RBL mast cells, where membrane-bound secretory vesicles dock and fuse (Deng et al., 2010; Jena, 2012). Studies on pancreatic acinar cells, whose secretion is also dependent on the *t*-SNARE SNAP-23, have revealed selective presence of SNAP-23 at the base of porosome, the site of secretory vesicle docking and fusion (Jena, 2012). We also could never find any LAMP translocating to plasma membrane after activation, ruling out full fusion between lysosome/granule and plasma membrane. By real time imaging, we have visualized SNAP-23 relocation to internal granule membranes including lysosomes that may be involved in homotypic fusion during compound exocytosis, as we have seen SNAP-23 in multiple granule fusion (homotypic fusion) just beneath the plasma membrane, upon activation. Granule-granule homotypic fusion is known to require SNARE rosette formation (Hammel and Meilijson, 2012). Another least-likely possibility is that it is the newly synthesized SNAP-23, which associates with the internal locations, and the older SNAP-23 are still associated with plasma membrane. But, in our experiments, the amount of total SNAP-23 was the same in resting, IgE-sensitized, and receptor crosslinked stages by western blot analysis of endogenous SNAP-23, or flow cytometric analysis of transfected GFP-SNAP-23. So, it is unlikely that in the short time span of 10 min after activation, newly synthesized SNAP-23 associates with internal locations and the older pool remains associated with the plasma membrane. In other studies involving secretory cells from adrenal medulla and pancreatic beta cells, *t*-SNARE SNAP-25 was shown to be important for sequential exocytosis and was supplied to primary granules by lateral diffusion from plasma membrane on stimulation (Kishimoto et al., 2006; Takahashi et al., 2004).

We have shown that SNAP-23 gets differentially phosphorylated (Hepp et al., 2005) in resting and activated MCs. In resting MCs, SNAP-23 is phosphorylated at Thr^102^, and this basal phosphorylation is not affected by activation. Ser^95^ and Ser^120^ are transiently phosphorylated immediately after activation, and mirror the kinetics of MC secretion. Both the MC secretion and induced SNAP-23 phosphorylation peak at 5-20 min, and show a decrease thereafter. Since all three major phosphorylation sites are in the linker region of SNAP-23, in close proximity to the conserved cysteine residues which may play an important role in anchoring SNAP-23 to membrane, we decided to study the role of SNAP-23 phosphorylation in its membrane associations in resting or activated MCs, respectively. Transfection, and expression of basal phosphorylation mutant SNAP-23 T102A, which cannot be phosphorylated, revealed that a large portion of SNAP-23 T102A accumulated in cytosol in resting MCs and the situation remained the same even after activation of MCs. Also, SNAP-23 T102A transfected MCs showed a 54% inhibition in exocytosis in comparison to controls. These results indicate that basal phosphorylation of SNAP-23 at Thr^102^ is important for the initial membrane association of SNAP-23. Due to the proximity of this site to conserved cysteine in the linker region of SNAP-23, it may function by affecting the palmitoylation of these cysteine residues. Or, it may affect initial association with membrane for palmitoylation, either by facilitating binding to some chaperone (Liu et al., 1996) or by changes in overall hydrophobicity (Polyansky and Zagrovic, 2012). Previous studies have indicated that the first association of similar proteins with membrane may be by some mechanism other than palmitoylation (Dunphy and Linder, 1998). For example, members of the Src family of tyrosine kinases or G protein α i1 subunit are cotranslationally myristoylated, and this helps in rapid membrane association (van't Hof and Resh, 1997) bringing them in close proximity of cellular palmitoyltransferases localized to intracellular membranes (Berthiaume and Resh, 1995; Dunphy et al., 1996; Liu et al., 1996). SNAP-23 is not myristoylated, leaving unresolved the mechanism by which SNAP-23 initially associates with membranes. SNAP-25, the key *t*-SNARE in neuronal cells that mediates synaptic vesicle release, gets palmitoylated for its membrane association at steady-state level in neuroendocrine cells, but its initial plasma membrane association depends on interaction with Syntaxin 1 while still in cytosol (Vogel et al., 2000). Hence some similar mechanism may be facilitated by Thr^102^ phosphorylation in SNAP-23 in MCs for efficient initial association with plasma membrane.

Further, when the inducible phosphorylation sites Ser^95^ and Ser^120^ of SNAP-23 were mutated to S95A/S120A for phospho-negative, and S95D/S120D for phospho-mimetic mutants, respectively, the dynamic regulated changes in SNAP-23 subcellular localization on MC activation were completely compromised. Membrane/cytosol fractionation and microscopy studies revealed that the phospho-negative SNAP-23 S95A/S120A mutant was able to dissociate from plasma membrane on activation as more of it ended up in cytosol in comparison to control, but unable to associate with internal LAMP-3-positive membranes. Hence, transient-induced phosphorylation of SNAP-23 at Ser^95^ and Ser^120^ seems to be important for association of SNAP-23 with internal lysosomal membranes. This conclusion was further validated by subcellular localization studies on the transfected phospho-mimetic SNAP-23 S95D/S120D mutant in resting and activated MCs. In both stages, the SNAP23 S95D/S120D mutant shows a higher colocalization with LAMP-3 containing internal membranes. Previously, in other studies, phosphorylation of claudin 1 (French et al., 2009) and beta-catenin (Qian et al., 2014) has been shown to regulate their subcellular localization and functions. Likewise, we have found that phosphorylation regulates membrane relocation of SNAP-23 during MC exocytosis. That means induced phosphorylations are mediating the relocation and internal membrane association of SNAP-23 to mediate the granule-granule fusion during MC mediator release. The present study reveals, for the first time, that induced phosphorylation of SNAP-23 has a role in dynamic changes in subcellular localizations of SNAP-23 in MCs undergoing degranulation. Mutations in phospho sites lead to a partial inhibition in exocytosis. So, maybe the induced phosphorylation of SNAP-23 is involved in bringing SNAP-23 to the right location, enabling it to participate in granule-granule fusion, which is an important step in compound exocytosis. The phosphomimetic mutant of SNAP-23 shows good association with internal LAMP-positive membranes, but its overall association with membranes is significantly lower than that of SNAP-23 wild type. So, more than 50% of this mutant remains displaced to cytosol, and hence fails to reach the right locations. These results indicate that the transient nature of phosphorylation is very important. The dephosphorylation following the phosphorylation may be important for recycling of SNAREs by priming or recycling of SNAP-23 back to plasma membrane (Hepp et al., 2005; Puri et al., 2003). As the SNAP23 S95D/S120D mutant is unable to show this dephosphorylated stage it is either stuck on internal membranes or in the cytosol, and hence causes a significant inhibition in exocytosis.

**Fig. 7.**
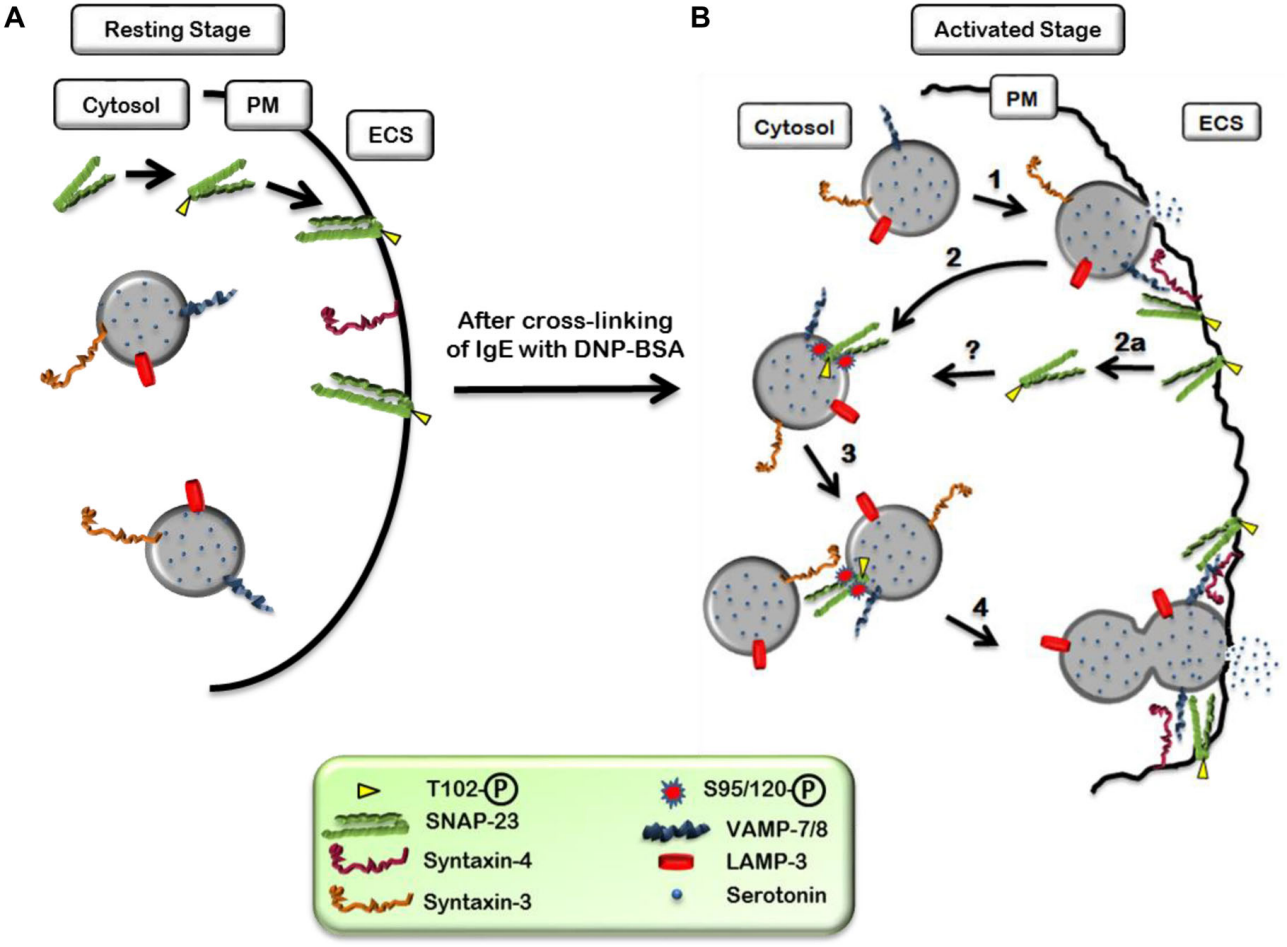
**Schematic representation of relocation of SNAP-23 during regulated exocytosis of MCs.** (A) In resting or IgE-sensitized MCs, SNAP-23 is synthesized in cytosol and phosphorylated at Thr^102^ which is important for its initial plasma membrane association. (B) After 10 min of the cross-linking of IgE with DNAP-BSA, SNAP-23 relocates from plasma membrane to internal organelle membrane by two possible mechanisms (1, 2/2a) and gets phosphorylated at Ser^95^ and Ser^120^ positions. This relocated SNAP-23 may mediate granule-granule fusion (3) and subsequently compound exocytosis from MCs (4).

On the basis of our findings we present a model for the molecular mechanisms of MC degranulation regulated by SNAP-23 phosphorylation (Fig. 7). Based on our results we hypothesize a dynamic pathway of SNAP-23 functioning that is represented by a schematic diagram (Fig. 7). Our earlier studies indicated localization of Syntaxin-3 and vesicle associated membrane protein (VAMP)-7/8 on LAMP-3-positive granules in MCs (Puri et al., 2003; Puri and Roche, 2008). So, SNAP-23 could be the *t*-SNARE counterpart in the ternary complex (where it is still present in phosphorylated state) for the secretory granule (SG)-SG fusion to occur. During physiological trigger, SNAP-23 relocation may be required for homotypic fusion of SGs followed by compound exocytosis in MCs. Until now there were no clear reports regarding molecules that are associated in the ternary SNARE complexes that mediate homotypic fusion in MCs. To our knowledge this is the first report showing SNAP-23 relocation to internal membranes in activated MCs during exocytosis under physiological conditions, and a role for SNAP-23 basal and induced phosphorylations in its dynamic membrane associations. Though detailed molecular mechanisms remain to be determined, we have clearly shown the dynamic nature of SNAP-23 membrane association may be to regulate different fusion steps in activated MCs leading to discharge of various inflammatory mediators.

## MATERIALS AND METHODS

### Cell culture

Rat basophilic leukemia mast cells [RBL-2H3, a kind gift from Dr Paul A Roche (NIH, Bethesda, MD, USA)] were maintained in equal parts minimal essential medium (Sigma, MO, USA) and Iscove's medium (Gibco, Life Technologies, Grand Island, NY, USA) containing 20% FBS (Gibco), 25 mM HEPES (Sigma), and 120 μg ml^−1^ gentamicin (RBL complete medium) as described (Hepp et al., 2005). Cells were maintained as sub-confluent monolayers at 37°C in a humidified atmosphere containing 5% CO2 and passaged with trypsin.

### Plasmids

pCMV-FLAG-Rat SNAP-23 wild type, pCMV-FLAG-Rat SNAP-23 T102A, pCMV-FLAG-Rat SNAP-23 95A/120A, and pCMV-FLAG-Rat SNAP-23 95D/120D were a kind gift from Dr Paul A Roche (NIH, Bethesda, MD, USA). The cDNA encoding full length wild-type SNAP-23 and its phosphomutants were subcloned from pCMV-FLAG-Rat SNAP-23 wild type into EGFP-C2 plasmid (#6083-1) (Clontech, CA, USA) by using *Eco*RI and *Apa*I restriction sites to generate amino terminal GFP-tagged protein (called GFP-SNAP-23). The integrity of subcloned plasmids was confirmed by sequencing from GCC Biotech and SciGenom Labs Pvt Ltd., India.

### Antibodies

Polyclonal rabbit anti-serum recognizing the SNAP-23 carboxyl terminus was a gift from Dr Paul A Roche (NIH, Bethesda, MD, USA). Anti–DNP IgE (clone TIB 142) was obtained from the American Type Culture Collection (Manassas, VA, USA). Mouse anti-CD63/LAMP 3 monoclonal antibody (mAb) AD1 (# 551458, 1:100), Mouse Anti-Rat trans-Golgi network (TGN) 38 antibody (# 610899, 1:100) and Mouse Anti-Golgi matrix protein (GM) 130 antibody (# 610822, 1:100) (BD Biosciences, San Diego, CA, USA), and anti-GFP rabbit mAb from Clontech were used in this study. Mouse anti serotonin antibody (# M0758, 1:50) was from Dako (Carpinteria, CA, USA). Alexa dye-conjugated secondary antibodies were obtained from Molecular Probes (Eugene, OR, USA) (anti Rb Alexa 546: A11035; anti mouse Alexa 546: A11030; anti mouse Alexa 488: A11001 dilution 1:500). Lysotracker Red (# L7528) was from Molecular Probes. Anti-Rabbit Protein A-HRP conjugated antibody (# 7300-05, 1:7000, Southern Biotech, Birmingham, AL, USA) and anti-mouse IgG-HRP conjugated antibody ((# 1031-05, 1:7000, Southern Biotech) were used. Antibody recognizing Syntaxin-3 (# ab133750, 1:800) (rabbit monoclonal) was obtained from Abcam (UK).

### Transfection of RBL cells

Transfection of RBL cells was performed as described earlier (Puri et al., 2003) with some modifications. Briefly, exponentially growing RBL cells (10×10^6^/0.5 ml serum-free RBL media) were transfected by electroporation (320 mV, 950 μF) with 20 μg DNA. Immediately after electroporation, the cells were plated in RBL complete medium and analyzed 24 h later. For microscopy, transfected cells were plated on coverslips and then treated as per requirement.

### Stimulation of RBL cell exocytosis in transfected RBL cells

RBL cells were transfected by electroporation with human growth hormone (2 µg) expression vector together with empty GFP vector or GFP-SNAP-23 wild type or phospho mutant (20 µg). After 4-5 h culture, the cells were sensitized with IgE and after 16-18 h they were mock-stimulated or stimulated with DNP-BSA as described before (Hepp et al., 2005). The amount of human growth hormone released into the medium or remaining cell associated was determined using a human growth hormone enzyme-linked immunosorbent assay (Roche Diagnostics Corp.) as described previously (Puri et al., 2003). For quantitative experiments, statistical analyses were carried out by using a Student's *t*-test. Results were considered significant when a *P* value of less than 0.05 was obtained.

### Confocal microscopy

RBL cells with or without transfection were seeded on 10 mm diameter coverslips. For indirect immunofluorescence analysis, the cells were either fixed with 4% paraformaldehyde (PFA) in PBS for 30 min and excess paraformaldehyde quenched with 50 mM NH4Cl in PBS or with cold methanol at −20°C for 4 min. After washing, the fixed cells were permeabilized with 1% IGEPAL (Sigma, MO, USA) in the presence of 3% normal goat serum (Sigma) and 0.05% saponin (SD Fine Chem. Limited, Boisar, India) in PBS. The cells were then incubated with 3% normal goat serum and 0.05% saponin in PBS for 1 h at RT to prevent nonspecific protein binding. Primary Abs diluted in the same buffer was added to the cells, and incubation was conducted for 1 h at room temperature (RT). After washing, the cells were incubated for 30 min in the presence of secondary goat Abs conjugated to Alexa Fluor 546 (red) (Molecular Probes). As a control, samples were stained with an irrelevant antibody and no staining was observed in the respective channel for all confocal fluorescence microscopy experiments.

Confocal images were collected with Olympus Fluoview FV1000 microscope at 100× magnification (sometimes with 2× zoom) with an optical slice thickness of 1.0 μm. Image Z-stacks were collected through the depth of the cell using 0.4 μm step size. Colocalization analysis was done for each plane of the individual image stacks using the colocalization analysis feature of the Fluoview software Ver.1.7a (Olympus). Briefly, individual channels were thresholded to include the structures of interest; regions of interest were then drawn to encompass the structures, resulting in scatter plots being generated and colocalization coefficients calculated. The colocalization coefficients represent colocalization in the green channel with respect to the red channel. Single images were exported from the Fluoview software Version 1.7a Software and organized into figures using Microsoft PowerPoint 2007.

### Real time imaging of GFP SNAP-23 expressing cells

RBL cells were transfected with EGFP-SNAP-23 plasmid, plated in a 3.5 cm culture dish and sensitized with anti-DNP IgE in RBL complete medium overnight at 37°C. Next day these were incubated with Lysotracker Red [a dye which stains the lysosomes and secretory granules as well in live cells (Marchini-Alves et al., 2012)] for 2 h in RBL complete medium. After washing with phenol red free RPMI medium they were either mock stimulated or stimulated with 100 ng ml^−1^ DNP-BSA and observed by a live cell imager Andor Spinning Disk Confocal microscope (Nikon Eclipse TiE, Software-Andor iQ 2.7) in 5% CO2 chamber at 37°C. The movies were captured 2 min after the addition of allergen and continued for 10 min. At least five movies were captured in the above manner for each separate experiment. All the images were analyzed by NIS element AR ver4.

### Membrane-cytosol fractionation

Membrane-cytosol fractionation was done as described earlier (Hepp et al., 2005). Briefly, transfected RBL mast cells were harvested after 24 h and resuspended in hypotonic buffer (10 mM Tris, 1 mM KCl, 1 mM EGTA, 0.5 mM MgCl2, pH 7.4) containing protease inhibitors (5 mM iodoacetamide, 50 mM PMSF, and 0.1 mM TLCK) and phosphatase inhibitors (5 mM EDTA, 5 mM EGTA, 50 mM NaF, 10 mM Na4P2O7, and 1 mM Na3VO4). They were then disrupted by repeated passage of cells through a 30.5 gauge syringe. Nuclei and unbroken cells were removed by centrifugation at 1000×***g*** and the post-nuclear supernatant was subjected to centrifugation at 100,000×***g*** for 1 h at 4°C to isolate membrane (pellet) and cytosol (supernatant). The membrane pellet and cytosolic supernatant were brought to the same volume in hypotonic buffer and each was adjusted to a final concentration of 1% Triton X-100. Equal portions of each fraction were analyzed by SDS-PAGE and immunoblotting.

### SDS-polyacrylamide gel electrophoresis and immunoblotting

RBL cell lysates and membrane-cytosol fractions were boiled in β-mercaptoethanol containing sample buffer and proteins were separated in 12.5% SDS-polyacrylamide gel. Immunoblotting was performed with polyclonal SNAP-23 C-terminus antibody or GFP antibody as previously described (Puri et al., 2003). As secondary antibodies anti-Rabbit Protein A-HRP and anti-mouse IgG-HRP were used. For immunoblotting separated proteins were transferred to 0.2 μ PVDF membrane (Bio-Rad, USA) and visualized by ECL using Immobilon Western Chemiluminescence HRP substrate (Millipore, MA, USA). Band intensity was determined by Spot Denso (AlphaEaseFC software, Alpha Innotech). For quantitative experiments, statistical analyses were carried out by using a Student's *t-*test in one-tailed distribution. Results were considered significant when a *P* value of less than 0.05 was obtained.
